# Hollow oaks and beetle functional diversity: Significance of surroundings extends beyond taxonomy

**DOI:** 10.1002/ece3.5940

**Published:** 2020-01-08

**Authors:** Ross Wetherbee, Tone Birkemoe, Olav Skarpaas, Anne Sverdrup‐Thygeson

**Affiliations:** ^1^ Faculty of Environmental Sciences and Natural Resource Management Norwegian University of Life Sciences Aas Norway; ^2^ Natural History Museum University of Oslo Oslo Norway; ^3^ Norwegian Institute for Nature Research (NINA) Oslo Norway

**Keywords:** biodiversity and ecosystem functioning, coleoptera, functional diversity, invertebrate conservation, *Quercus* spp., saproxylic beetles, veteran trees

## Abstract

Veteran hollow oaks (*Quercus* spp.) are keystone structures hosting high insect diversity but are declining in numbers due to intensification of land use and the abandonment of traditional management. The loss of this vital habitat is resulting in a reduction of biodiversity, and this likely has consequences for ecosystem functioning, especially if functional diversity is reduced. A considerable amount of research has been done on predictors of beetle taxonomic diversity in veteran oaks, but predictors of functional diversity have remained largely unexplored. The aim of this study was to establish whether the features and surroundings of veteran oaks are related to functional diversity within three functional groups of beetles (decomposers, predators, and flower visitors) and determine whether species richness and functional diversity within the groups are dependent on the same predictors. Sampling was carried out intermittently between 2004 and 2011 on 61 veteran oaks in Southern Norway. Of the 876 beetle species that were collected, 359 were determined to be decomposers, 284 were predators, and 85 were flower visitors. Species richness and functional diversity in all groups were consistently higher in traps mounted on veteran oaks in forests than in open landscapes. However, additional predictors differed between groups, and for species richness and functional diversity. Decomposer species richness responded to tree vitality, while functional diversity responded to habitat connectivity, predator species richness responded to regrowth of shrubs while functional diversity responded to tree circumference, and flower visitor richness and functional diversity did not respond to any additional predictors. Previous studies have found that the features and surroundings of veteran oaks are important for conservation of taxonomic diversity, and the results from this study indicate that they are also important for functional diversity within multiple functional groups.

## Graphical abstract

Veteran hollow oaks (Quercus spp.) are keystone structures hosting high insect diversity. The aim of this study was to establish whether the features and surroundings of these trees are related to functional diversity of beetles within three functional groups (decomposers, predators, and flower visitors) and determine whether species richness and functional diversity within the groups are dependent on the same predictors. Previous studies have found that veteran oak's features and surroundings are important for conservation of taxonomic diversity, but the results from this study clearly indicate that they are also important for supporting multiple ecosystem functions.

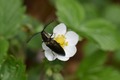

## 1 INTRODUCTION

Human activities are converting and degrading ecosystems at unprecedented levels (Newbold et al., 2015), and this has resulted in dramatic biodiversity loss in the last 50 years (Reid et al., 2005). This decline is predicted to continue or even accelerate in the coming years (Newbold et al., 2015). Biodiversity regulates ecosystem processes such as energy and matter fluxes over space and time, and maintenance of life on earth depends on these processes (Laureto, Cianciaruso, & Samia, 2015). Therefore, this is a greater problem than a reduction in the number of species, as there is robust evidence that biodiversity has a positive effect on ecosystem functioning (Brose & Hillebrand, 2016; Cardinale et al., 2012; Laureto et al., 2015).

Although there is a link between biodiversity and ecosystem functioning, the relationship is not straightforward (Lefcheck & Duffy, 2015). Ecosystem functioning is more closely related to species trait diversity than to species richness (Heemsbergen et al., 2004; Lefcheck & Duffy, 2015). A diversity of effect traits, that is, an aspect of an organism's morphology, physiology, phenology, or behavior with an effect of ecosystem processes (Reiss, Bridle, Montoya, & Woodward, 2009), promotes multiple ecosystem processes and makes these processes more resistant and resilient to change (Diaz & Cabido, 2001; Folke et al., 2004; Grime et al., 1997; Reiss et al., 2009; Tilman, Isbell, & Cowles, 2014). Studies on functional diversity have the potential to reveal patterns that studies on taxonomic diversity are unable to detect and allow for the possibility to draw inferences about effects on ecosystem functioning (Cadotte, Carscadden, & Mirotchnick, 2011; Hooper et al., 2005; Laureto et al., 2015; Mouillot et al., 2013). Therefore, identifying drivers of functional diversity is a crucial step in the process of predicting and possibly mitigating reduction of ecosystem functioning.

Veteran trees are keystone structures in forest, agricultural landscapes, and urban areas (Lindenmayer, Laurance, & Franklin, 2012; Müller, Jarzabek‐Müller, Bussler, & Gossner, 2013; Parmain & Bouget, 2018). The size and age of veteran trees, as well as their coarse bark, structurally complex canopies, and sheltered nutrient‐rich cavities provide features that support high biodiversity (Lindenmayer et al., 2012; Parmain & Bouget, 2018). However, veteran trees are in decline globally as a result of intensification of land use and the abandonment of traditional management (Siitonen & Ranius, 2015). Veteran oaks (*Quercus* spp.) are especially important for biodiversity in Northern European forest (Siitonen & Ranius, 2015). The community of invertebrates associated with veteran oaks is of high management interest and is important for decomposition and nutrient recycling, and also has high diversity of predators and flower visitors (Ranius, Niklasson, & Berg, 2009b; Sverdrup‐Thygeson, Skarpaas, & Ødegaard, 2010).

A considerable body of work has gone into identifying the main predictors of taxonomic diversity of veteran oak beetle communities, and it is well established that the features and surroundings of the tree influence the community (Micó, 2018). The main factors that have been shown to affect species richness are tree size, regrowth around the tree, sun exposure and temperature, amount and diversity of dead wood, height of the hollow, hollow and wood mold volume, habitat isolation, and stand maturity (Gossner et al., 2016; Gough, Birkemoe, & Sverdrup‐Thygeson, 2014; Micó, García‐López, Sánchez, Juárez, & Galante, 2015; Miklín et al., 2018; Parmain & Bouget, 2018; Pilskog, Birkemoe, Framstad, & Sverdrup‐Thygeson, 2016; Ranius & Jansson, 2000; Ranius, Niklasson, & Berg, 2009a; Sverdrup‐Thygeson et al., 2010). Furthermore, there has been a noted distinction between beetle communities in veteran trees in forests and similar trees in open landscapes (Parmain & Bouget, 2018; Sverdrup‐Thygeson, Skarpaas, Blumentrath, Birkemoe, & Evju, 2017; Sverdrup‐Thygeson et al., 2010). Research has also found that oak‐dependent saproxylic species respond to substrate density across different spatial scales (Bergman, Jansson, Claesson, Palmer, & Milberg, 2012; Sverdrup‐Thygeson et al., 2017).

Although recent studies have begun to focus on traits in addition to taxonomic diversity, this work has been primarily focused on trophic levels (Pilskog et al., 2016), patterns associated with specialists and generalists (Sverdrup‐Thygeson et al., 2017), and the response of traits to management strategies (Gossner et al., 2013; Janssen, Fuhr, Cateau, Nusillard, & Bouget, 2017). To our knowledge, no work has been done on veteran oak beetle community effect traits, and it is unknown how the features and surroundings of veteran oaks will affect this community's functional diversity.

The aim of this study was to establish how the features and surroundings of veteran oaks are related to functional diversity within three functional groups (decomposers, predators, and flower visitors) and determine whether species richness and functional diversity within the groups are dependent on the same set of predictors. We chose traits that were relevant for their respective ecosystem function and therefore considered different effect traits within each group. The advantage of this approach is that it is possible to observe patters that are relevant for both conservation and ecosystem functioning (Brose & Hillebrand, 2016; Fountain‐Jones, Baker, & Jordan, 2015; Laureto et al., 2015; Mayfield et al., 2010; Tilman et al., 2014).

Since the functional groups depend on different resources and we considered different traits within the groups, we expected different predictors to be important for the groups. We expected diversity in decomposers to respond to predictors associated with the amount and diversity of dead wood (e.g., forest cover, tree vitality, and presence of other hollow trees), predator diversity to respond to predictors associated with the amount and diversity of prey (e.g., tree size, which increase the number and diversity of microhabitats), and flower visitor diversity to the abundance and richness of flowering plants (e.g., light and openness, and regrowth around the trees).

## 2 MATERIAL AND METHODS

Sampling was carried out on 61 veteran hollow oaks (*Quercus robur* and *Q. petraea*) at 27 sites in the main distribution of oaks in Southern Norway (Figure 4). Sites were originally selected based on forest inventories of hollow oaks from the forestry sector and the municipalities. Some of the sites had cluster of up to five trees, while others had just one tree. The data set used in this study was collected intermittently between 2004 and 2011 and is part of a long‐term study of veteran oaks and the National Program for Surveying and Monitoring Biodiversity in Norway (ARKO, 2011). A veteran hollow oak was defined with national management regulations, as a tree of at least 95cm circumference with a visible cavity in the trunk (Lovdata, 2011).

Sampling was conducted with flight intercept traps: 20 × 40 cm windows with a funnel below leading to a vial containing ethylene glycol, water (4:1 mixture), and a drop of detergent used as a surfactant. It is important to note that flight intercept traps are the least selective method of sampling tree hollows and are biased toward flying beetles species (Micó, 2018). They are activity traps that capture dispersing insects and have been extensively used to measure veteran tree insect communities (Micó, 2018; Parmain & Bouget, 2018; Sverdrup‐Thygeson et al., 2017, 2010). Although flight interception traps detect activity, the beetle communities caught in this type of trap hanging from a dead or hollow tree have been shown to reflect species associated with the dead wood resource (Isaksen, 2015; Sverdrup‐Thygeson & Birkemoe, 2009). It has also been found that the same proportion of dead wood associated insects has been collected in flight intercept traps as in other sampling techniques, such as emergence traps (Birkemoe & Sverdrup‐Thygeson, 2015), and the same responses have been identified along gradients (Müller et al., 2015). Furthermore, the presence of exit holes on the dead wood source has been correlated with species caught in flight intercept traps. Thus, they represent a widely used and efficient way of estimating beetles in dead wood. In this study, we were interested in the entire veteran oak beetle community, not just the communities within the hollows. Each tree was therefore sampled with two traps: one directly in front of the hollow and one hanging in the canopy. Traps were activated in May and emptied once a month until the middle of August. Due to the methodology of the Program for Surveying and Monitoring Biodiversity in Norway (ARKO, 2011), some trees were sampled over multiple years: 20 trees were sampled for 1 year, 27 trees were sampled for 4 years, and 14 trees were samples for 5 years.

**Figure 1:**
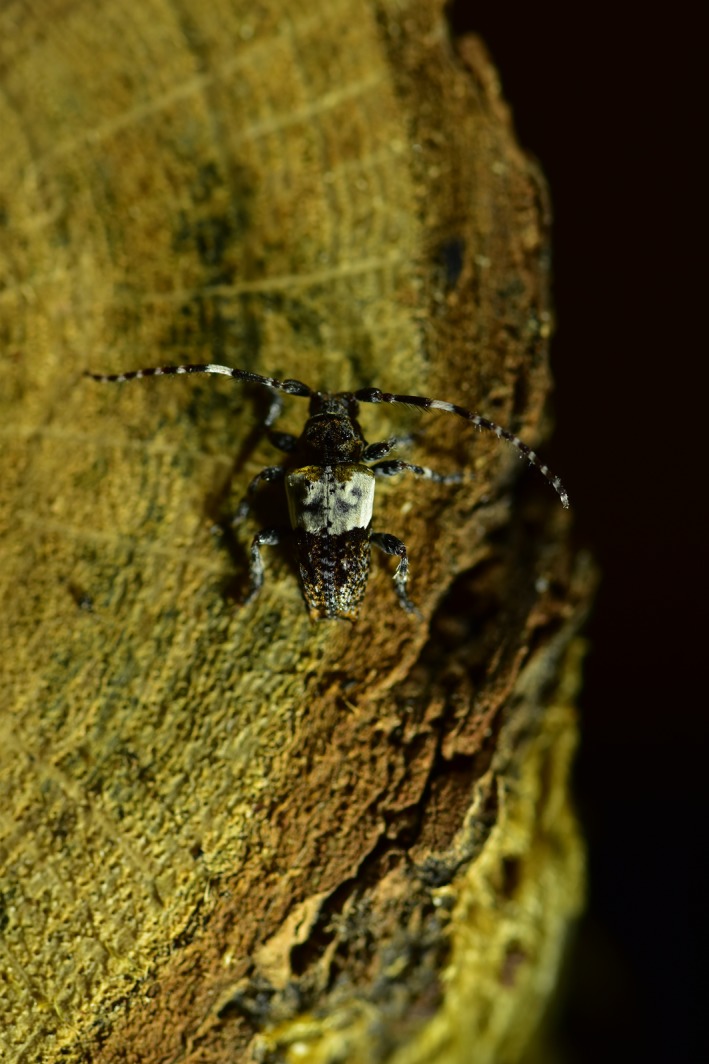
Example a beetle that is a decomposer: *Pogonocherus hispidulus*

**Figure 2:**
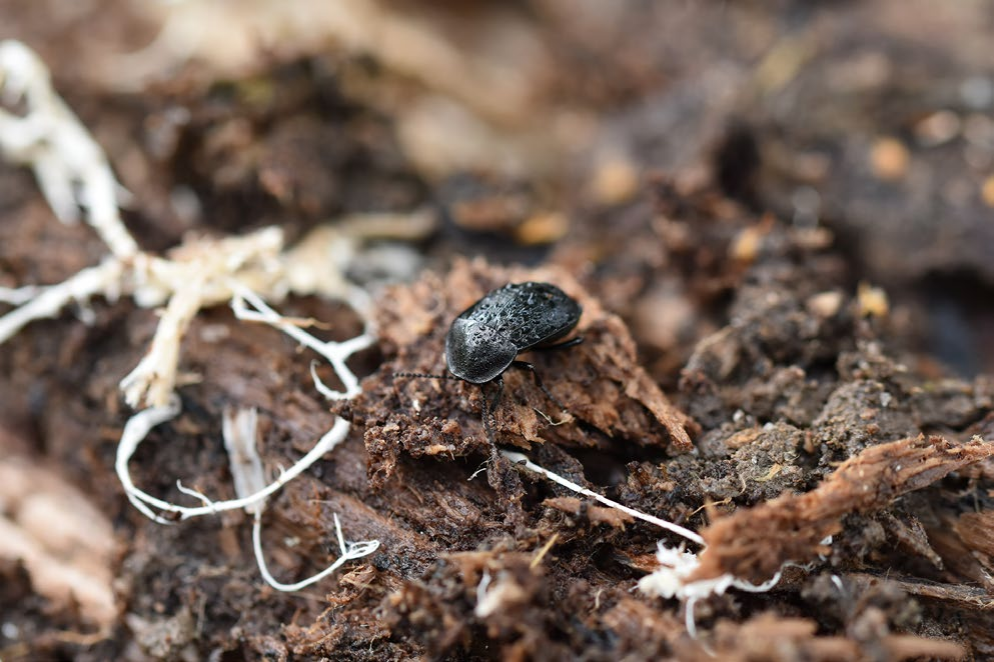
Example of a predatory beetle*: Phosphuga atrata*

**Figure 3:**
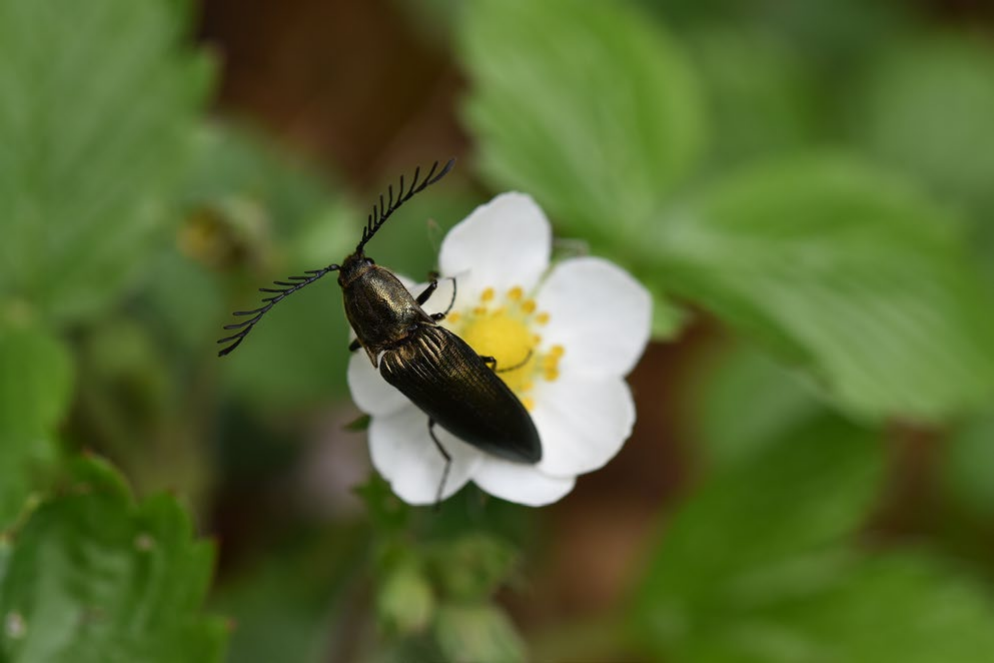
Example of a flower‐visiting beetle: *Ctenicera pectinicornis*

All beetles were identified to the species level following the taxonomy of The Norwegian Biodiversity Information Centre (NBIC 2018). The species were subsequently divided into the three functional groups: decomposers, predators, and flower visitors based on published literature (Figures 1, 2, 3, Appendix II). Species were assigned to the groups based on both adult and larval diet and were therefore not mutually exclusive. Species that we could not find trait information for or did not fit into at least one of the three functional groups were removed from the data set. This came to 30% of the total number of sampled species (262 species). Important effect traits related to decomposition, predation, and pollination were identified based on previous studies (Gossner et al., 2013; Pilskog et al., 2016; Seibold et al., 2015; Sverdrup‐Thygeson et al., 2017) and protocols for selecting insect functional traits (Fountain‐Jones et al., 2015; Moretti et al., 2017).

We used body size (measured as length in mm) for all functional groups, because it is closely linked to many life‐history traits such as life span and dispersal ability, and it influences the amount and composition of resources used by the organism (Fountain‐Jones et al., 2015, Gillespie, Birkemoe, & Sverdrup‐Thygeson, 2017, Moretti et al., 2017). For decomposers, we also used wood diameter and decay stage preference, because a diversity of preferred habitats will aid the decomposition process (Gossner et al., 2013); and if the species was a known polypore visitor, because recent research suggests that beetles visiting polypores play a role in dispersing fungal spores (Birkemoe, Jacobsen, Sverdrup‐Thygeson, & Biedermann, 2018). For predators and flower visitors, we used peak activity date, because having species active throughout the season will increase phenological overlap with prey species and flowering plants (Gillespie et al. 2017, Moretti et al., 2017). In addition, we included the morphological traits of relative eye size for predators and body shape for flower visitors. Eye size is linked to prey recognition as well as hunting strategy (Fountain‐Jones et al., 2015; Moretti et al., 2017), and body shape may influence how the species interacts with floral architecture (Patt, Hamilton, & Lashomb 1997, Fountain‐Jones et al., 2015). Trait information for beetle species was primarily collected from literature, but some trait information was calculated from available material (Table 1).

**Table 1:** A summary of species richness and functional diversity for each of the functional groups, along with the traits used to calculate functional diversity

Functional group	Species richness	Functional diversity	Traits	Type/unit of measurement	Collection source
Decomposer	Total: 359 Mean: 23 Max: 52 Min: 4	Mean:0.20 Max:0.27 Min:0.02	Body length	Continuous: mm	Literature
			Polypore visitor	Binomial	Literature
			Wood diameter preference	Continuous: developed by Gossner et al. (2013)	Literature
			Decay preference	Continuous: developed by Gossner et al. (2013)	Literature
Predator	Total: 284 Mean: 15.8 Max: 39 Min: 1	Mean:0.08 Max:0.14 Min:0.03	Body length	Continuous: mm	Literature
			Eye size	Continuous: eye size divide by length	Photogrammetric analysis
			Peak activity date	Continuous: year days	Literature and predictions from GBIF data
Flower visitor	Total: 85 Mean: 6.1 Max: 25 Min: 1	Mean: 0.13 Max: 0.25 Min: 0.02	Body length	Continuous: mm	Literature
			Body shape	Continuous: width at widest place divide by length	Photogrammetric analysis
			Peak activity date	Continuous: year days	Literature and predictions from GBIF data

Functional dispersion was used as a measure of functional diversity and was calculated from different traits within each of the functional groups. Important effect traits related to the respective ecosystem function were identified based on previous studies and protocols for selecting insect functional traits. Species richness and functional diversity were calculated for each veteran oak for every year of sampling (*N* = 202, 61 trees sampled intermittently from 2004 to 2011).

**Figure 4:**
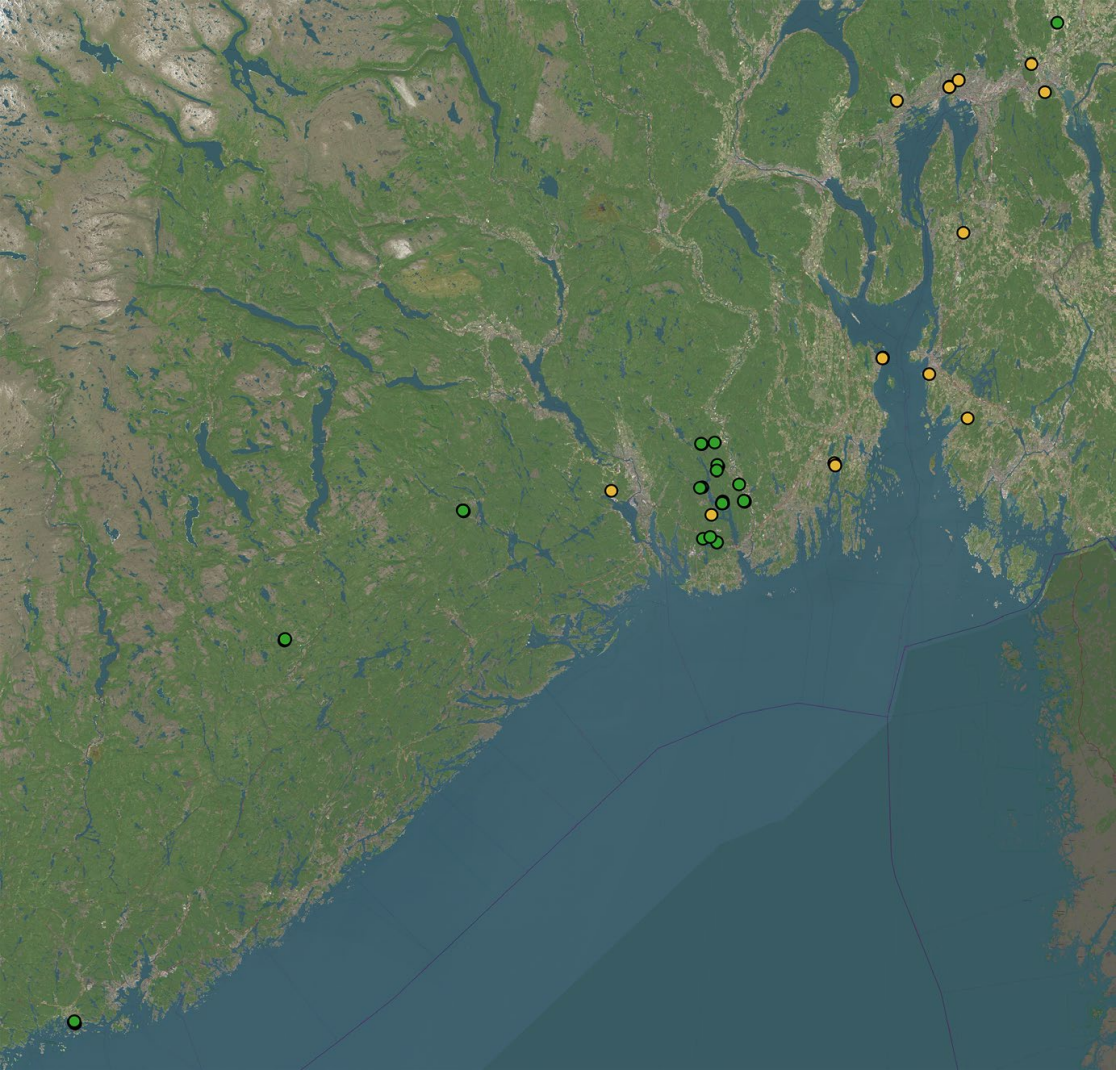
Sampling was carried out on 61 hollow oaks *(Quercus sp.)* in forest (green circles) and open landscapes (yellow circles) at 27 sites located in the main distribution of oaks in Southern Norway. Data were collected intermittently between 2004 and 2011 as part of the National Program for Surveying and Monitoring Biodiversity in Norway (ARKO 2009)

Functional diversity was calculated based on all traits within each of the functional groups. Since functional diversity indices are sensitive to missing trait information, we verified that at least 80% of all species in the functional groups had trait information (Majekova et al. 2016). All species that were excluded as a result of lack of data were rare in the data set (less than five individuals). We chose to use functional dispersion (FDis) to measure functional diversity because it accounts for species abundances, species richness has limited effect on it (Figure 4 in Appendix I), and it can be calculated for multiple traits and can include both continuous and categorical measurements (Laliberte & Legendre, 2010). FDis is a measure of dispersion in trait space, is calculated as the mean distance of species to the centroid of the community and is weighted by abundances (Laliberte & Legendre, 2010).

Since all traits within the functional groups had Pearson correlation coefficient less than 0.7, we considered them to add independent contributions and chose not to weigh any of the traits when calculating the FDis (Appendix I). FDis was also calculated for all traits individually and then tested for correlations with the tree and landscape variables in order to verify that there were no counteracting effects (Appendix I). We calculated species richness and FDis with the *dbFD* function in the “FD” package (Laliberte & Legendre, 2010; R Development Core Team, 2017). FDis was calculated using a Gower dissimilarity matrix and the "cailliez" correction method (Cailliez, 1983; Lalibert & Legendre, 2010).

Following a protocol set by Sverdrup‐Thygeson et al. (2017), we classified veteran oaks into two habitat classes based on the dominating habitat with in a 50 m radius of the tree. Oaks were located either in forests or in open landscapes (Table 2). Forests trees were primarily in multiple‐use managed forests, which contained a diversity of other tree species and decaying wood. Trees in open landscapes were either in parks or in agricultural landscapes and were surrounded by fields or gardens with low amounts of dead wood (forest trees = 38 and open landscape trees = 23). Variables that described the tree and its surrounding were collected at the time of sampling (Table 2) and were chosen because they have been shown to have an effect in other oak–beetle studies (Parmain & Bouget, 2018; Pilskog et al., 2016; Ranius & Jansson, 2002; Sverdrup‐Thygeson et al., 2017, 2010). We also included a measure of the connectivity of the study tree to other veteran oaks at different spatial scales (Table 2). Connectivity was estimated with a published distribution model for veteran oaks (Skarpaas, Blumentrath, Evju, & Sverdrup‐Thygeson, 2017) by summing the predicted probabilities of occurrence of veteran oaks for all pixels in radii of 0.5‐25 km of the focal tree. The model was based on a stratified random sample of veteran oak presences and absences across the oak distribution in Southern Norway and geographic and land cover predictors that represent a mixture of ecological and anthropogenic factors.

**Table 2:** Variables that described the veteran oak and its surrounding landscape and were used to predict species richness and functional diversity of beetles captured around the tree

Variable	Type	Measurement	Reference
Tree circumference	Continuous	Tree circumference at breast height in cm. Natural logarithm taken	Sverdrup‐Thygeson et al. (2010)
Light availability	Continuous	Slope*cos(Aspect−45)	Stage (1976), Stage and Salas (2006)
Vitality	Categorical (2 levels)	Healthy: 20% or more living canopy Senescent: <20% living canopy	Sverdrup‐Thygeson et al. (2017)
Regrowth	Categorical (3 levels)	Open: no regrowth around oak Shrubs: low vegetation, shrubs or small trees Trees: trees with height similar to the focal tree	Sverdrup‐Thygeson et al. (2017)
Connectivity (8 variables)	Continuous	Predicted number of hollow oaks within 0.5 km, 1 km, 2 km, 3 km, 4 km, 5 km, 10 km, and 25 km of focal tree	Skarpaas et al. (2017)
Habitat class	Categorical (2 levels)	Forest: tree situated in a forest Open landscape: tree in either a park or agricultural landscape	Sverdrup‐Thygeson et al. (2010)
Tree cover density (3 variables)	Continuous	20 m: the percent of the 20 m pixel where focal tree is located that is covered by forest. 100 m: the percent of 20 m pixels covered by forests within 100 m radius of focal tree. Standard deviation: the standard deviation of the percent of 20 m pixels covered by forests within 100 m radius of focal tree	CLMS (2012 & 15)

Trees were either located in forest (*n* = 38) or open landscapes (*n* = 23).

In order to incorporate substrate density at different spatial scales, we used Copernicus tree cover density maps with 20 m resolution to calculate the tree cover density (TCD) at the 20 m scale, the 100 m scale, and the standard deviation at the 100 m scale for all trees (CLMS, 2012, & 15). We chose these two scales so that we would have one that was finer and one that was courser than the habitat class variable (50 m), which was obtained in the field. This approach gave us the possibility to consider continuous landscape variables measured at different scales. Since sampling ended in 2011, we used the 2012 maps when possible, but when there was missing data, we included data from 2015 maps (Table 2). However, preliminary analysis indicated that there was very little overlap in TCD between the habitat classes. Furthermore, when the response variables were separated into habitat classes, TCD was not a significant predictor (Appendix I). We concluded that habitat class was a better variable at describing the landscape and at predicting how functional groups respond to it. We therefore dropped TCD from further analysis.

Since trees were sampled repeatedly over a varying number of years, we included year and tree as crossed random effects in the models to adjust for repeated sampling of the trees and year‐to‐year variation. We also considered using site in the random effect structure but found that it did not improve the models, so it was dropped from the analysis. We used linear mixed models with Gaussian error distribution to model the influence of the tree and landscape variables on FDis since it was approximately normally distributed in our data set. We also used generalized linear mixed models with the same random effect structure and Poisson error distribution to model the effects on species richness. Prior to statistical analysis, we followed the steps for data exploration outlined by Zuur, Leno and Elphick (2010). All continuous predictor variables were scaled by subtracting the mean and dividing by the standard deviation of the data matrix using the *scale* base function in R (version 3.4.0). In order to avoid collinearity, no model contained variables with Pearson correlation coefficient of 0.5 or higher, and no model had a variation inflation factor (VIF) greater than three (Zuur, Ieno, Walker, Saveliev, & Smith, 2009). The best model was chosen with backward model selection based on Akaike information criterion (AIC) (Zuur et al., 2009). We carried out model selection separately for correlated variables and subsequently compared the final models using AIC to determine the optimal model (Arnold, Sabom, Nisbet, & Hatch, 2006). The residuals of the final linear models were checked for normality and homogeneity, and the generalized linear models were checked for overdispersion. Models were also checked for influential observations, as well as patterns between the residuals and all potential predictor variables, sampling date, and geographic location (Zuur et al., 2009).

Statistical analysis was carried out in R version 3.4.0 (R Development Core Team, 2017). Linear mixed models were created using the *lmer* function, and generalized linear mixed models were created with the *glmer* function in the “lme4” package (Bates, Machler, & B. B. and and S. Walker., 2015). The R‐squared and P values were calculated for the models with packages “MuMIn” and “lmerTest” (Barton, 2018; Kuznetsova, Brockhoff, & Christensen, 2017). The following packages were also used for data manipulation, statistical analysis, and graphical visualization: “Lattice” (Sarkar, 2008), “e1071”(Meyer, Dimitriadou, Hornik, Leisch, & W. A., 2018), “nlme” (Pinheiro, Bates, & D. S., and S. D., 2018), “predictmeans” (Luo, Ganesh, & K. J., 2018), “ggplot2” (Wickham, 2016), “Hmisc” (Harrel & Dupont, 2018), and “dplyr” (Wickham, F. R., H. L., & K. Müller., 2018).

## 3 RESULTS

In total, 31,617 individuals from 876 species were collected between 2004 and 2011. Of these 27,314 individuals from 614 species could be included in at least one of the functional groups and had available trait information. A total of 359 species were determined to be decomposers, 284 were determined to be predators, and 85 were flower visitors. The groups were not mutually exclusive, so 45 species were determined to be both decomposers and predators, 44 were decomposers and flower visitors, 25 were predators and flower visitors, and three species were included in all groups. The mean decomposer species richness per tree and year was 23 (min–max: 4–52); mean predator species richness was 16 (1–39); and mean flower visitor species richness was 6 (1–25).

**Figure 5:**
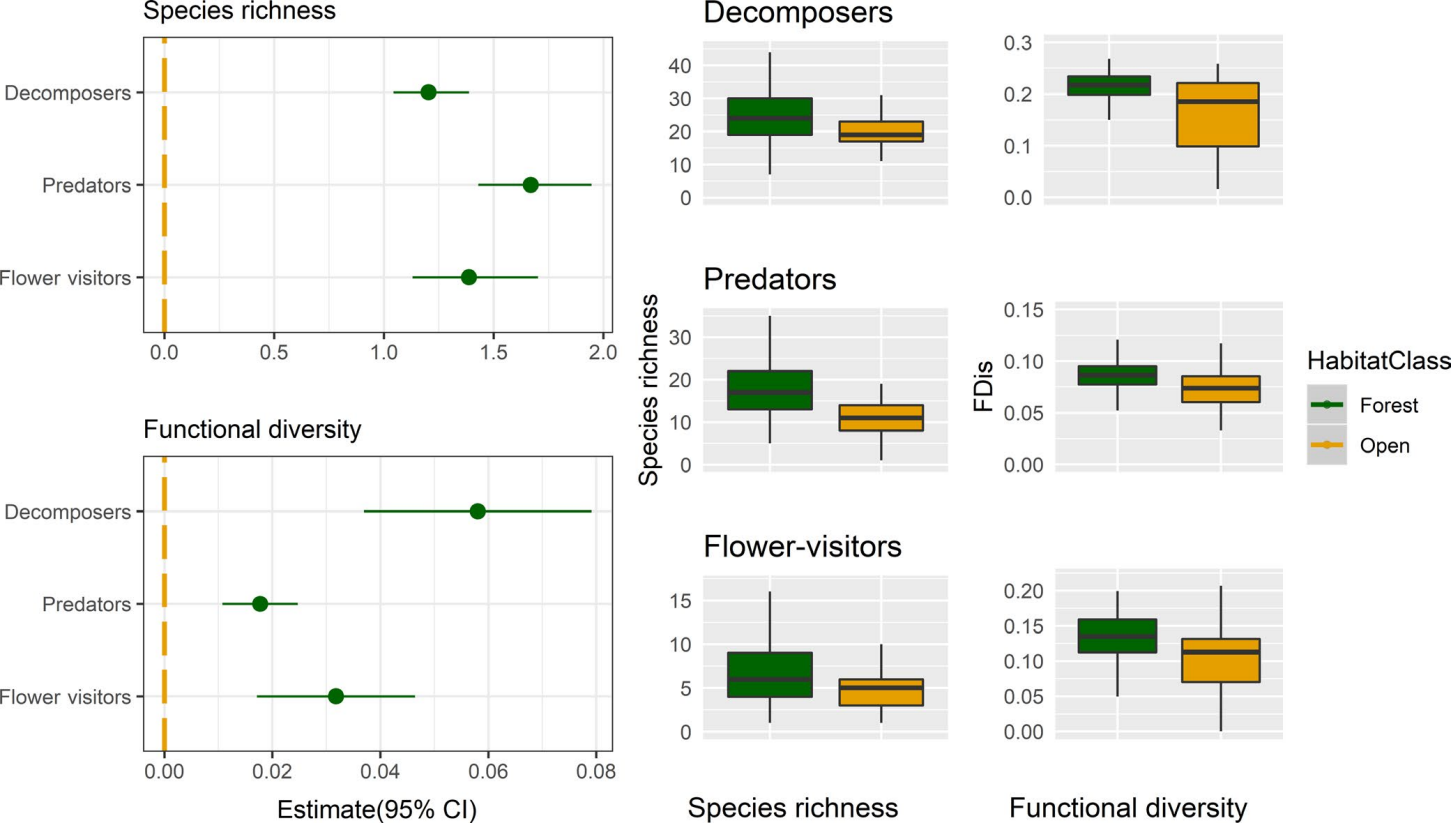
Species richness and functional diversity in all groups were significantly higher in traps mounted on veteran oaks surrounded by forest (green) than those in open landscapes (yellow). The magnitude of this effect varied between functional groups, and for species richness and functional diversity. The plots on the left are partial regression coefficients (with 95% CI) for the effect of forest surroundings on species richness (top) and functional diversity (bottom). The boxplot on the right is observations of species richness (middle plots), and functional diversity (right plots) in forests and open landscapes for each of the functional groups. The boxplots show the median, first, and third quartiles, with whisker that extend 1.5 times the interquartile range. In all plots, the veteran oaks in open landscapes are colored yellow and in forests are green

Species richness and functional diversity in all groups (decomposers, predators, and flower visitors) was consistently higher in traps mounted on veteran oaks surrounded by forest than those in open landscapes (Table 3 and Figure 5). The magnitude of this effect, however, varied between functional groups, and for species richness and functional diversity. For functional diversity, forest surroundings had the strongest effect on decomposers and a weaker effect on predators and flower visitors, and for species richness, this trend was reversed (Figure 5).

**Table 3:** Results from the best generalized linear mixed model (species richness) and linear mixed models (functional diversity) with year and tree as random effects.

Species richness	Decomposers β	Predators β	Flower visitors β
intercept	3.136***	2.894***	1.866***
Habitat class (forest)	0.186***	0.512***	0.328**
Tree vitality (Senescent)	0.265**		
Regrowth (shrubs)		0.207*	
Regrowth (trees)		−0.037	
AIC	1,391	1,213	1,060
R^2^ fixed	0.14	0.32	0.07
R^2^ full	0.52	0.62	0.33
Functional diversity			
intercept	0.211***	0.087***	0.132***
Habitat class (forest)	0.058***	0.018***	0.032**
Connectivity: Habitat class (forest)	0.003		
Connectivity: Habitat class (open)	0.026*		
Tree circumference		0.004*	
AIC	−660	−1032	−672
R^2^ fixed	0.22	0.15	0.10
R^2^ full	0.60	0.31	0.21

The models were used to test the effects of the tree and landscape variables on the functional group's species richness and functional diversity. Species richness and functional diversity were calculated for each veteran oak for every year of sampling (*N* = 202, 61 trees sampled intermittently from 2004 to 2011). The best models were identified with backward model selection based on AIC. All continuous predictor variables were scaled by subtracting the mean and dividing by the standard deviation of the data matrix (Significance codes: ***<0.001, **<0.01, *<0.05).

### 3.1 Decomposers

**Figure 6:**
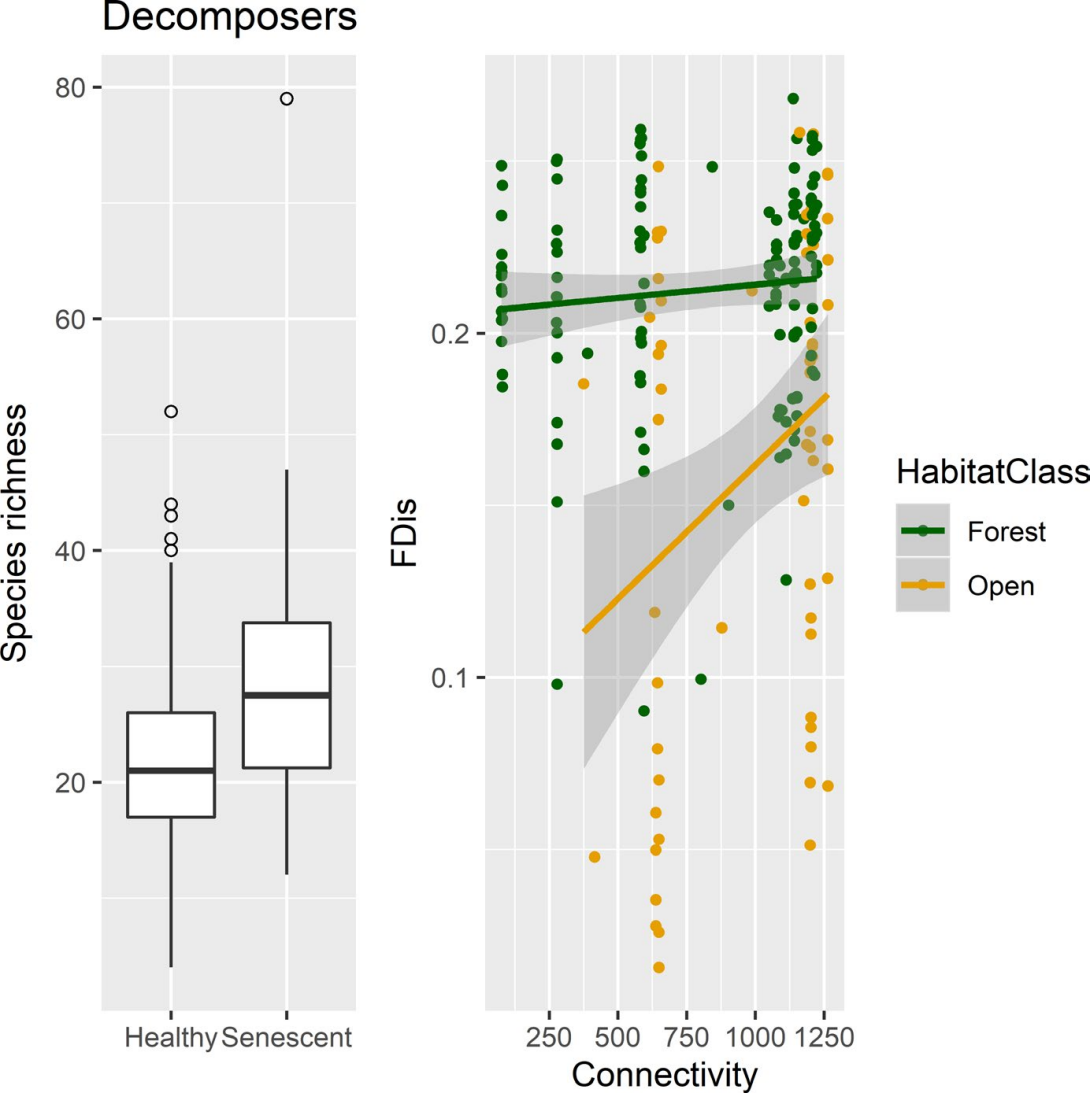
*Left plot:* Boxplot of species richness and health of the tree. Decomposer species richness was significantly higher in traps mounted on senescent trees than healthy ones. The plot shows the median, first, and third quartiles, with whisker that extend 1.5 times the interquartile range, and observations beyond the end of the whiskers as points. *Right plot:* Decomposer functional diversity (FDis, y‐axis) was significantly higher in traps mounted on trees in forests (green) than in open landscapes (yellow), and connectivity (x‐axis, measured as the number of predicted veteran oaks within a 5 km radius of the focal tree) had a significate positive effect in open landscapes but no effect in forests. Regression lines are the results from a linear mixed model that predicted FDis with year and tree as random effects, and the gray area around the lines is the 95% confidence interval. Values in the figure have been back transformed, but for the analysis all continuous predictor variables were scaled by subtracting the mean and dividing by the standard deviation of the data matrix

In addition to the difference between forest and open landscape, we found that decomposer species richness responded to the vitality of the tree, being higher in senescent trees (*β* = 0.265, *p* = .002, Table 3 and Figure 6). Functional diversity, meanwhile, was affected by connectivity in open landscapes (*β* = 0.026, *p* = .041, Table 3 and Figure 6). Connectivity was significant at 2‐10 km scale in open landscapes, and the strongest effect and least amount of error around the estimate was at the 5 km scale (*β* = 0.026, *p* = .407), but there was no significant effect of connectivity in forests (*β* = 0.003, *p* = .693, Table 3 and Figure 6). The model predicting species richness explained less of the fraction of the variation than the model predicting functional diversity (R^2^ fixed = 0.14 and 0.22, Table 3).

### 3.2 Predators

**Figure 7:**
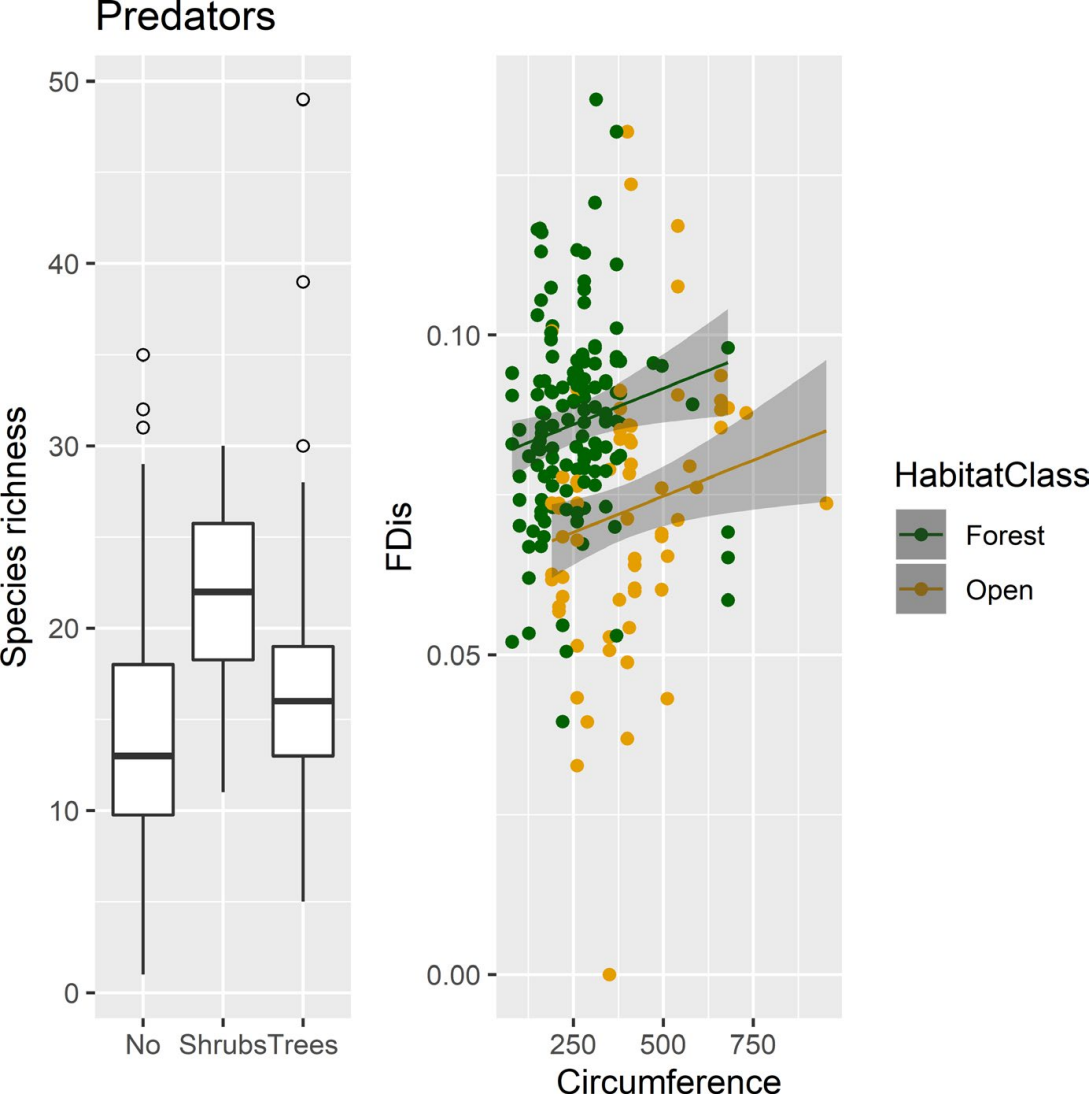
*Left plot:* Boxplot of species richness and regrowth around the tree. Predator species richness was significantly higher when there was regrowth of shrubs around the trees. The plot shows the median, first, and third quartiles, with whisker that extend 1.5 times the interquartile range, and observations beyond the end of the whiskers as points. *Right plot:* Predator functional diversity (FDis, y‐axis) was significantly higher in traps mounted on trees in forests (green) than in open landscapes (yellow), and tree circumference had a significate positive effect. Regression lines are the results from a linear mixed model that predicted FDis with year and tree as random effects, and the gray area around the lines is the 95% confidence interval. Values in the figure have been back transformed, but for the analysis all continuous predictor variables were scaled by subtracting the mean and dividing by the standard deviation of the data matrix and tree circumference was log‐transformed to reduce the effect of extreme observations

Independent of whether the tree was in a forest or open landscape, predator species richness increased with shrub regrowth (*β* = 0.207, *p* = .003, Table 3 and Figure 7). Functional diversity, on the other hand, increased with tree circumference (*β* = 0.004, *p* = .014, Table 3 and Figure 7). The model that predicted species richness explained twice as much of the variation as the model predicting functional diversity (R^2^ = 0.31 and 0.15, Table 3).

### 3.3 Flower visitors

The only significant predictor of flower visitor species richness and functional diversity was whether the tree was in a forest or in an open landscape, with greater values being measured in traps mounted on trees in forests (Table 3). In addition, the fixed part of the models only explained a small fraction of the variation in species richness and functional diversity (R^2^ = 0.07 and R^2^ = 0.10, Table 3).

## 4 DISCUSSION

In this study, we found that the features and surroundings of the veteran oaks influenced species richness and functional diversity of beetles, whether classified as decomposers, predators, or flower visitors. There was one trend that held for all groups: When the trees were situated in forest rather than open landscapes, both species richness and functional diversity were higher. Apart from this, the additional predictors differed between groups, and for species richness and functional diversity. This indicates that focusing on only species richness or functional diversity may result in missing predictors that are relevant for conservation and ecosystem functioning.

### 4.1 Forest versus open landscapes, across functional groups

The result that species richness was higher in traps mounted on trees in forest than in open landscapes for all groups matches the general trends that have been identified for veteran oak beetle communities in Northern Europe (Bergman et al., 2012; Pilskog et al., 2016; Sverdrup‐Thygeson et al., 2017, 2010), but the result that functional diversity was higher in all groups is a new finding. Both results, however, contrast to research done in France, where Parmain and Bouget (2018) found more rare species in veteran trees in open landscapes than veteran trees in forests and no difference between total species richness. It is possible that the higher number of rare species would result in higher functional diversity (Jain et al., 2014; Mouillot et al., 2013; Soliveres et al., 2016; Violle et al., 2017). If that was the case, it would be an intriguing contrast to our results, but this currently remains unverified.

Another interesting result is that there was some indication that decomposers were responding differently than the other groups to forests surroundings. Decomposer species richness was the least affected by forest surroundings, while functional diversity was the most. This indicates that more often than the other functional groups, the additional decomposer species in forests had trait combinations that were not present in open landscapes. Understanding how species are filtered from communities according to their traits remains a fundamental question in community ecology (McGill et al., 2007; Sutherland et al., 2013). One explanation for our results is that the decomposer communities captured in traps mounted on trees in forests were more influenced by surrounding because their traits were tightly linked to amount and diversity of dead wood, which was clearly higher and more diverse in forests. On the other hand, the resources that drove predator and flower visitor functional diversity may not have been so tightly linked to the differences between forest and open landscapes. However, it is also possible that these results were an artifact of our trait choices (we considered a different set of traits for each group) and therefore should not be overstated.

### 4.2 Decomposers

In addition to the results above, we found that reduced connectivity had a negative effect on decomposer functional diversity, but forests surroundings modulated this effect. We found that habitat connectivity in open landscaped increased functional diversity, but it did not have an effect in forests. Communities within isolated trees may have a strong selective pressure for traits that can be sustained by the veteran tree. This likely results in environmental filtering and reduced dispersion of the community's traits (Córdova‐Tapia, Hernández‐Marroquín, & Zambrano, 2018; Mason, Bello, Mouillot, Pavoine, & Dray, 2013; Mason et al., 2012).

Effects of habitat connectivity have also been observed on species richness and abundances of veteran oak‐dependent beetles (Pilskog et al., 2016; Sverdrup‐Thygeson et al., 2017). Our results add to these findings by indicating that isolation may cause functional homogenization of decomposers. This is potentially problematic, as a recent study has experimentally linked isolation with reduced functional diversity and shown that it has a negative impact on decomposition rates (Mestre, Jansson, & Ranius, 2018).

Decomposer species richness did not respond to connectivity, but instead increased with decreasing tree vitality. This finding matched our predictions as tree vitality relates to the amount of dead wood available in the veteran tree. However, other studies found mixed results when considering the effects of veteran oak vitality on taxonomic diversity of saproxylic beetles. Vitality of veteran oaks did not have an effect on rare and threatened beetles or on generalist saproxylic species richness (Sverdrup‐Thygeson et al., 2017, 2010), but did affect species richness of oak specialist and abundances within diverse feeding guilds (Pilskog et al., 2016; Sverdrup‐Thygeson et al., 2017).

In this study, the effect of tree vitality on species richness was not paralleled by an effect on functional diversity. The mechanism for this discrepancy remains unknown, but these results indicate that a large patch of dead wood might ensure high species richness, which may not necessarily correspond to higher rates of decomposition. However, functional redundancy of communities can make the ecosystem function they support more resilient to species loss and environmental changes (Fonseca & Ganade, 2001; Hooper et al., 2005).

### 4.3 Predators

Predator functional diversity increased with tree size. As tree size increases, so does structural heterogeneity, which increases the number of microhabitats available for potential prey species (Parmain & Bouget, 2018; Ranius & Jansson, 2000). Many of the habitats, such as course bark, moss covering the tree trunk, sap flows, dendrotelms (water‐filled hollows), and vertebrate nests, are beneficial to species other than decomposing or flower‐visiting beetles and may be related to higher numbers and greater diversity of prey species. Thus, we predicted that predator diversity would increase with tree size, and our prediction was supported for functional diversity but not species richness. Earlier research has found that total saproxylic beetle species richness increased with tree size (Gough et al., 2014; Parmain & Bouget, 2018; Ranius & Jansson, 2000; Sverdrup‐Thygeson et al., 2010), but the only study focusing on predators as a group found no such correlation for species richness (Pilskog et al., 2016).

We found that predator species richness instead increased with regrowth of shrubs. There is some precedent for this result, as Pilskog et al. (2016) found that regrowth influenced predatory beetles around veteran oaks. Furthermore, the family of Carabidae, which are primarily predators and an important family in our data set, has been shown to have a link to plant taxonomy and traits (Brose, 2003; Niemelä, Koivula, & Kotze, 2006; Niemelä, Spence, & Spence, 1992; Pakeman & Stockan, 2014), but it should be pointed out that our trapping methods were not ideal for sampling this group as they are biased toward flight dispersing beetles (Micó, 2018). Nevertheless, regrowth of shrubs around the veteran trees likely increases habitat heterogeneity and this has been shown to have a positive effect on species diversity (Tews et al., 2004). Functional diversity, however, was not affected by regrowth, which may indicate that there is not a benefit of regrowth for biological control, as it has been found that an increase in functionally similar predators does not necessarily increase predation rates of invertebrate pests (Snyder, 2019; Straub & Snyder, 2006).

### 4.4 Flower visitors

In contrast to the other functional groups, no additional predictors of functional diversity or species richness were identified for flower visitors. Also, the best models that predicted flower visitor species richness and functional diversity explained only a small fraction of the variation. Clearly, major predictors of this group remain unknown. It is important to note that flower‐visiting activity is only applicable to adult beetles and that the pollen or nectar acquired is typically a supplementary diet. Of the 85 species of flower visitors, only 18 were not included among the decomposer and/or predators. It is likely that species within this group were responding differently to the predictors. Nevertheless, given the abundance and diversity of flower‐visiting beetles that this study found, it is likely that these communities contribute to pollination in the surrounding landscapes.

### 4.5 Conclusion and management implications

The importance of forests surrounding veteran trees has been demonstrated for the conservation of rare and threatened beetles (Sverdrup‐Thygeson et al., 2010), and our results indicate that surrounding forest cover is also important for supporting multiple ecosystem processes. We also suggest that managers should promote high densities of veteran oaks over large areas. Our results support the concept that these trees are not independent units and that veteran trees collectively add value across large spatial scales (Sverdrup‐Thygeson et al., 2017, 2010). Furthermore, large veteran trees provide a source of predators and are likely beneficial to pest control when the trees are near agricultural fields or in managed forests. Another possible benefit of veteran oaks for agriculture is pollination from the diverse flower‐visiting beetle communities. Our results indicate that management strategies can be implemented to support decomposition, predation, and flower visitation, and good practices have the potential to add value for conservation and ecosystem functioning.

In conclusion, a large body of work has shown that features and surroundings of veteran oaks are important for conservation of species diversity, and the results from this study indicate that they are also important for functional diversity within multiple functional groups. We also conclude that functional and taxonomic diversity respond to different drivers between and within the functional groups, so both measures should be considered as complimentary and potentially relevant for future research focused on conservation and ecosystem functioning.

## CONFLICT OF INTERESTS

The authors declare that they have no competing interests.

## AUTHORS' CONTRIBUTIONS

RTW contributed to the study design, analyzed the data, and wrote the first draft of the manuscript. TB contributed to the study design and critical review of the draft. OS contributed to the study design, supervised data analysis, and contributed critical review of the draft. AS was responsible for the data set, contributed to the study design, and critical review of the draft. All authors assisted in the writing of the manuscript and approve the final version.

## ETHICS APPROVAL AND CONSENT TO PARTICIPATE

This study complied with the appropriate institutional, national, and international guidelines.

## Data Availability

All data used in this study is deposited in DataverseNO (https://doi.org/10.18710/VF2FTT).
