# Supplementary material for: Hollow oaks and beetle functional diversity: Significance of surroundings extends beyond taxonomy
Source: Ecol Evol. 2020 Jan 8;10(2):819–31. doi: 10.1002/ece3.5940 (PMC6988526; doi:10.1002/ece3.5940)
Supplement: Supplementary file 1 [file ECE3-10-819-s001.docx]

**Appendix**

*Functional groups and traits*

Trait information for beetle species was primarily collected from literature, but some trait information was calculated from available material. Peak activity time was established by Gillespie et al. (2017) and was estimated using flight activity period data. For species that we did not have a measurement of peak activity time, we calculated one based on a linear model using GBIF data. We calculated the mean event date in the GBIF data and then used a linear model to predict peak flight date. We then used the model to make predictions for peak activity time for species that were missing in Gillespie et al. (2017) data (Figure 1). In the GBIF data, we only took observations from Norway that happened between March and September. We also removed species from the dataset that had fewer than 20 observations.

We also chose to include the morphological traits of relative eye size for predators and body shape for flower-visitors. We gathered photographs of the beetles from online databases. In order to obtain a relative measure of eye size, we measured each eye and length of the beetle using the program *imageJ*. We then dived the mean of the eye measurements by the length of the beetle. We followed a similar protocol to obtain a relative measure of shape. We measured the beetle at the widest place and its length. We then dived width at the widest place by length.

*Functional dispersion*

Before calculating the functional dispersion (FDis) of all the traits, we first calculated the Pearson correlation coefficient between the traits. Since all traits within the functional groups had a correlation coefficient less than 0.7, we considered them to add independent contributions, and chose not to weigh any of the traits when calculating the FDis (Figure 2). FDis was also calculated for all traits individually and then tested for correlations with the landscape and site variables, in order to verify that there were no counteracting effects (Table 2).

*Tree cover density*

Habitat class (weather the trees was in a forest or open landscape) and tree cover density at the 100m scale (TCD) had the strongest effect on both species richness and functional diversity in all the functional groups. However, preliminary analysis indicated that there was very little overlap in TCD between the habitat classes (Figure 3), and the effect of these variables was very similar: species richness and functional diversity increased with tree cover and from open landscapes to forests. However, when the response variables were separated into habitat classes, TCD was not a significant predictor (Table 3). We therefore, conclude that the primary effect is a result of differences in habitat class, and TCD also captured this difference. TCD was subsequently dropped from the analysis.


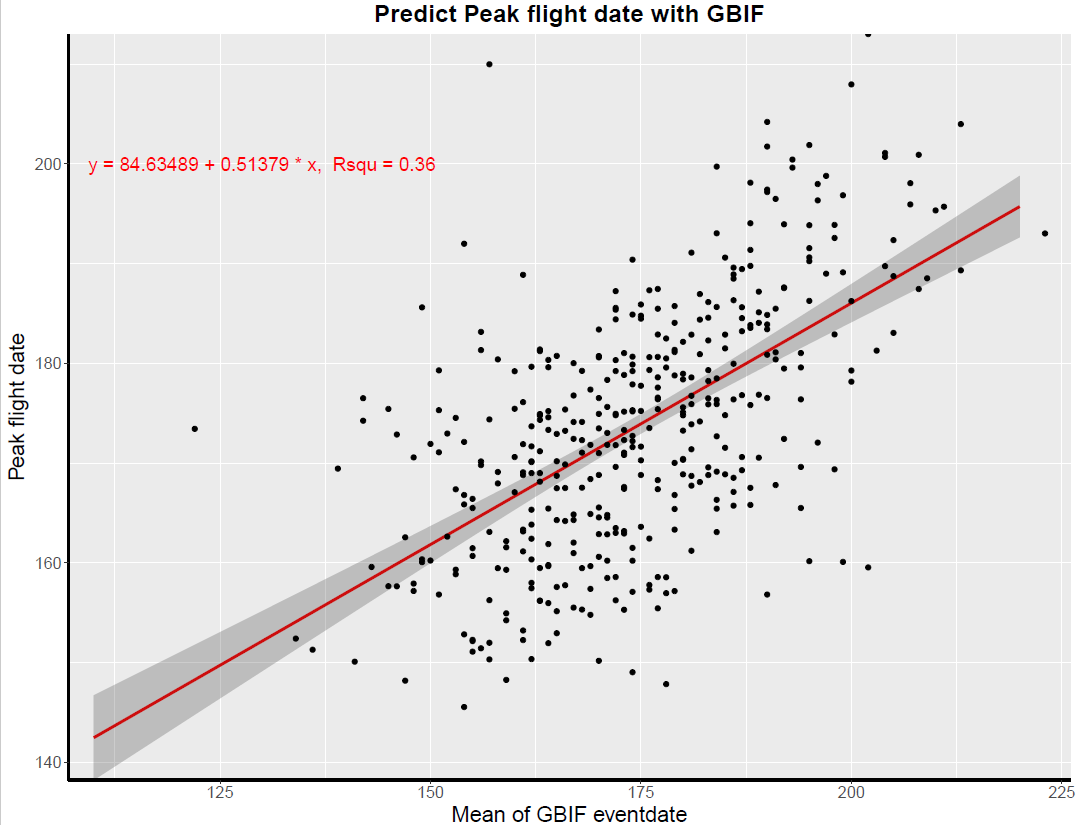


**Figure 1:** Results form a linear model that predicted Gillespie et al. (2017) measure of peak flight date with the mean event date from GBIF data. We only took observations from Norway that occurred between March and September, and removed species from the data that had fewer than 20 observations. We then used this model to predict the peak flight date for species that were not present in Gillespie et al. (2017) data.

**
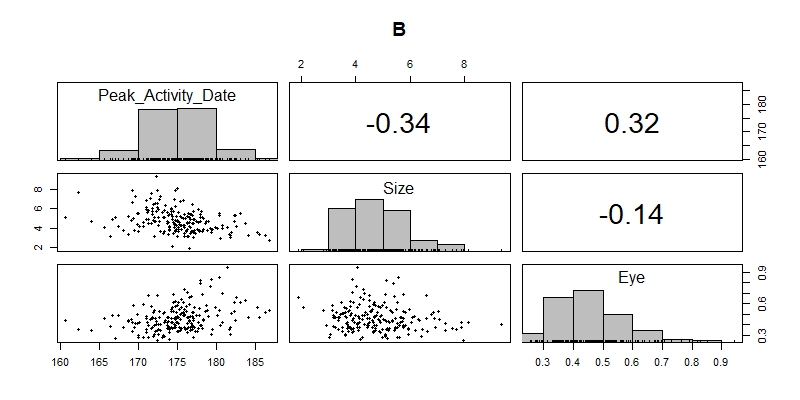

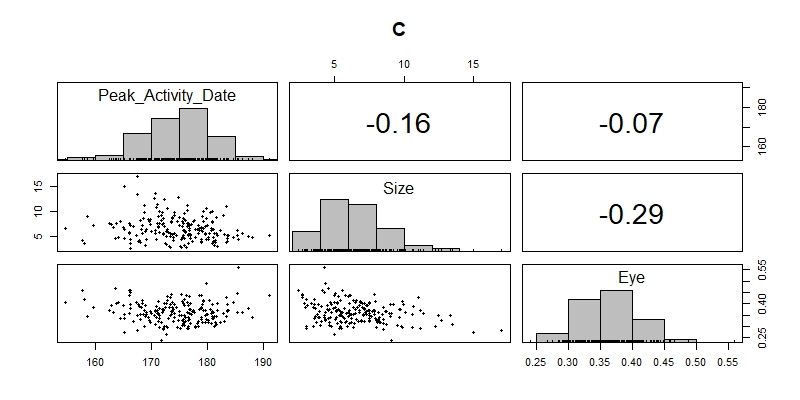

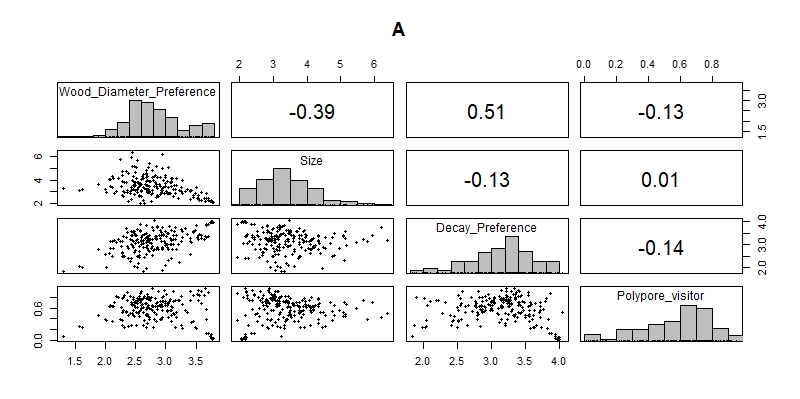
Figure 2:** Plots of the CWM of the traits for decomposer (plot A), predators (plot B) and flower-visitors (plot C). Plots below the diagonal are bivariate scatter plots, on the diagonal are histograms on the diagonal, and above the diagonal is the Pearson correlation coefficient.


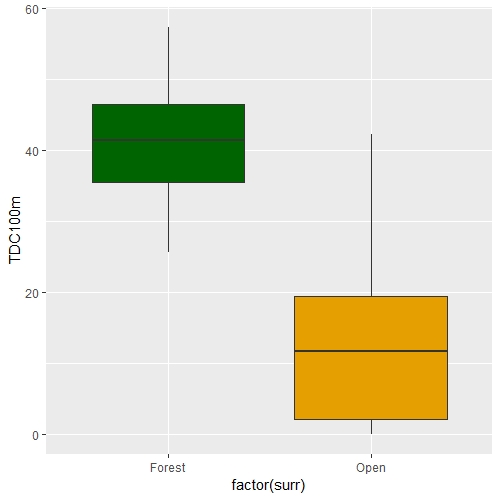


**Figure 3:** Boxplot of TCD at the 100m scale and Habitat class around the veteran tree. There was very little overlap in TCD between the habitat classes. The plot shows the median, first and third quartiles, with whisker that extend 1.5 times the inter-quartile range. Veteran oaks in open landscapes are colored yellow and in forests are green.


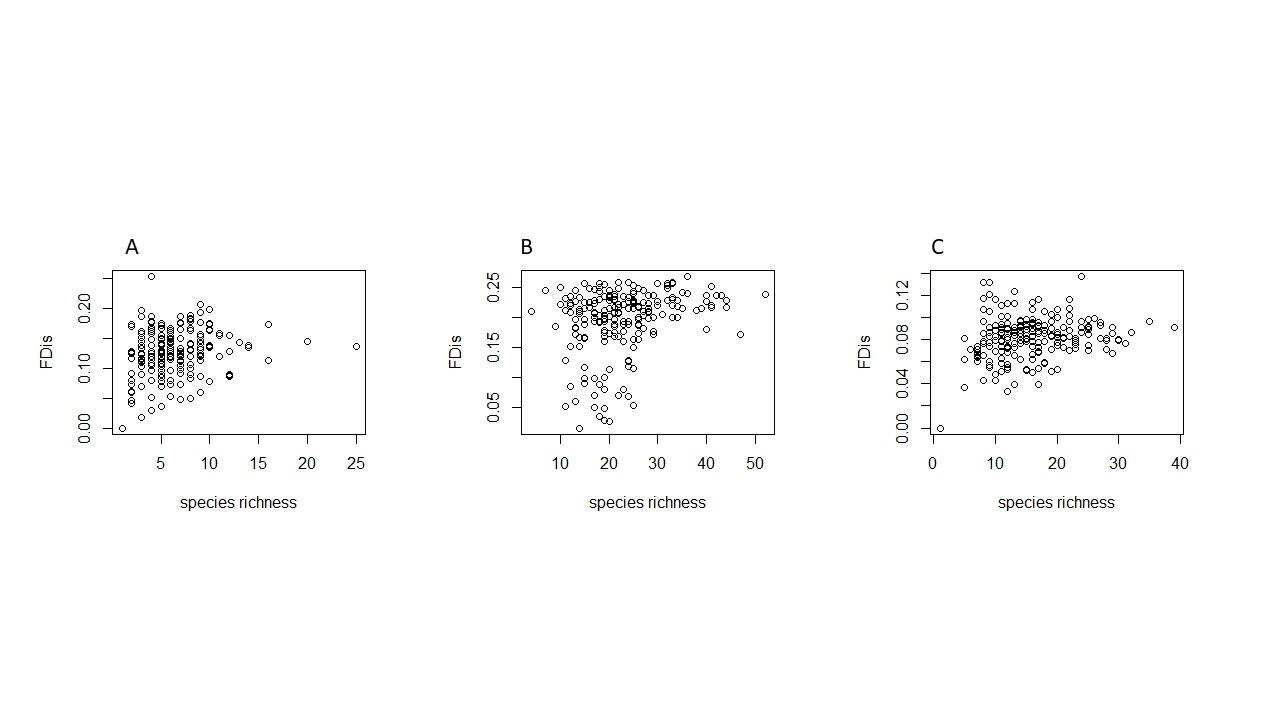
**Figure 4:** Scatter plot of FDis (y axis) and species richness (x axis) for predators (plot A), decomposers (plot B) and flower-visitors (plot C). The person correlation coefficient for predators was 0.17, for decomposer was 0.28, and for flower-visitor was 0.18.

**Table 1:** Pearson correlation coefficient between FDis for each trait and the landscape and site variables. FDis was calculated for each trait and for all the traits and tested for counteracting effects.

| **Decomposers**  **traits** | **Tree Circ.** | **Habitat class** | **Connectivity** | **Vitality** | **Tree form** | **Regrowth** | **Slope** | **Aspect** |
| --- | --- | --- | --- | --- | --- | --- | --- | --- |
| Size | -0.13 | -0.53 | >0.01 | 0.31 | 0.09 | 0.44 | 0.09 | 0.14 |
| Polypore visitor | -0.19 | -0.41 | >0.01 | 0.22 | 0.07 | 0.33 | 0.24 | 0.09 |
| Decay preference | - 0.28 | -0.36 | >0.01 | 0.19 | 0.25 | 0.22 | 0.34 | >0.01 |
| Wood Diameter  preference | - 0.29 | -0.25 | 0.11 | 0.10 | 0.24 | >0.01 | 0.29 | 0.18 |
| All | - 0.23 | - 0.45 | 0.01 | 0.24 | 0.14 | 0.34 | 0.29 | >0.01 |
| **Predators**  **traits** |  |  |  |  |  |  |  |  |
| Size | -0.15 | -0.35 | >0.01 | 0.17 | >0.01 | 0.16 | 0.27 | 0.08 |
| Activity time | 0.15 | -0.12 | >0.01 | -0.06 | -0.13 | >0.01 | >0.01 | >0.01 |
| Eye size | >0.01 | >0.01 | -0.12 | >0.01 | >0.01 | -0.17 | >0.01 | >0.01 |
| All | >0.01 | -0.33 | >0.01 | -0.09 | >0.01 | 0.16 | 0.16 | >0.01 |
| **Flower-visitor traits** |  |  |  |  |  |  |  |  |
| Size | >0.01 | -0.16 | >0.01 | >0.01 | >0.01 | 0.17 | 0.11 | 0.10 |
| Activity time | -0.14 | -0.33 | >0.01 | >0.01 | >0.01 | 0.14 | 0.19 | 0.34 |
| Body shape | >0.01 | -0.18 | >0.01 | >0.01 | >0.01 | 0.11 | 0.10 | 0.15 |
| All | >0.01 | -0.32 | >0.01 | >0.01 | >0.01 | 0.22 | 0.18 | 0.26 |

**Table 2:** Pearson correlation coefficient between community weighted mean (CWM) for each continuous trait and the landscape and site variables. CWM was calculated using the dbFD function in the ‘FD’ package (Laliberte and Legendre 2010, R Development Core Team 2017).

| **Decomposers**  **traits** | **Tree Circ.** | **Habitat class** | **Connectivity** | **Vitality** | **Tree form** | **Regrowth** | **Slope** | **Aspect** |
| --- | --- | --- | --- | --- | --- | --- | --- | --- |
| Size | -0.11 | -0.48 | 0.11 | 0.26 | 0.1 | 0.45 | 0.23 | 0.11 |
| Decay preference | 0.22 | >0.01 | >0.01 | >0.01 | -0.22 | >0.01 | >0.01 | >0.01 |
| Wood Diameter  preference | 0.34 | 0.45 | >0.01 | >0.01 | >0.01 | -0.3 | -0.2 | -0.11 |
| **Predators**  **traits** |  |  |  |  |  |  |  |  |
| Size | >0.01 | -0.16 | >0.01 | >0.01 | -0.15 | >0.01 | >0.01 | >0.01 |
| Activity time | >0.01 | -0.21 | 0.1 | >0.01 | >0.01 | 0.13 | 0.1 | >0.01 |
| Eye size | 0.13 | 0.3 | >0.01 | -0.11 | >0.01 | -0.22 | -0.22 | >0.01 |
| **Flower-visitor traits** |  |  |  |  |  |  |  |  |
| Size | >0.01 | -0.1 | >0.01 | >0.01 | >0.01 | 0.18 | >0.01 | 0.1 |
| Activity time | >0.01 | -0.11 | >0.01 | 0.1 | 0.12 | -0.14 | 0.16 | -0.1 |
| Body shape | >0.01 | >0.01 | 0.12 | >0.01 | 0.17 | >0.01 | >0.01 | >0.01 |

**Table 3:** Results from generalized linear mixed model (species richness) and linear mixed models (functional diversity) with year and tree as random effects. Species richness and functional diversity were calculated for each veteran oak for every year of sampling (N=203, 61 trees sampled intermittently from 2004 to 2011) and were predicted with an interaction between TCD and habitat class (Significance codes: 0 ‘***’, 0.001 ‘**’, 0.01 ‘*’).

| **Species richness** | **Decomposers**  β | **Predators**  β | **Flower-visitors**  β |
| --- | --- | --- | --- |
| intercept  TCD: Habitat class (open)  TCD: Habitat class (forest) | 2.945***  -0.002  0.006 | 2.904***  -0.002  -0.041 | 1.764***  0.006  0.002 |
|  |  |  |  |
| **Functional diversity** |  |  |  |
| intercept  TCD: Habitat class (open)  TCD: Habitat class (forest) | 0.228***  -0.001  -0.047 | 0.082***  -0.000  0.006 | 0.142  0.000  0.000 |
|  |  |  |  |
