# Supplementary material for: Hollow oaks and beetle functional diversity: Significance of surroundings extends beyond taxonomy
Source: Ecol Evol. 2020 Jan 8;10(2):819–31. doi: 10.1002/ece3.5940 (PMC6988526; doi:10.1002/ece3.5940)
Supplement: Supplementary file 3 [file ECE3-10-819-s003.docx]

**Appendix II Reference list**

Alekseev, M. A., E. E. Sinitsina, and S. Y. Chaika. 2006. Sensory organs of the antennae and mouthparts of beetle larvae (Coleoptera). Entomological Review **86**:638-648.

Anderson, J. M. 1972. Food and feeding of Notiophilus biguttatus F. (Coleoptera: Carabidae).

Audisio, P. A., P. Cornacchia, E. Gatti, S. Hardersen, L. Leseigneur, G. Nardi, F. Penati, and G. Platia. 2008. Selected beetle families in natural forests and Norway Spruce stands at Vincheto di Celarda Nature Reserve and the effects of conservation actions.*in* Reaearch on the natural heratige of Vincheto di Celarda and Val Tovenella, Belluno, Italy.

Austara, Ø., C. Cater, J. Eilenberg, G. Halldorsson, and S. Harding. 1997. Natural enemies of the green spruce aphid in spruce plantions in maritime North-West Europe. Icelandic Agricultural Sciences **11**:113-134.

Baker, F. A., D. W. French, H. M. Kulman, O. Davis, and R. C. Bright. 1985. Pollination of the eastern dwarf mistletoe. Canadian Journal of Forest Research **15**:708-714.

Baker, G. H. 1981. Notes on the Ecology of the Beetle Dascillus cervinus (Coleoptera: Dascillidae). The Irish Naturalists' Journal **20**:336-338.

Beal, R. S. 1998. Taxonomy and Biology of Nearctic Species of Anthrenus (Coleoptera: Dermestidae). Transactions of the American Entomological Society (1890-) **124**:271-332.

Bouget, C., B. Nusillard, X. Pineau, and C. Ricou. 2012. Effect of deadwood position on saproxylic beetles in temperate forests and conservation interest of oak snags. Insect Conservation and Diversity **5**:264-278.

Bozdoğan, H., M. Erbey, and H. A. Aksoy. 2016. Total amount of protein, lipid and carbohydrate of some adult species belong to curculionidae family (Coleoptera: Curculionidae) Journal of Entomology and Zoology Studies **4**:242-248.

Buckland, P. 2007. The development and implementation of software for palaeoenvironmental and palaeoclimatological research: the Bugs Coleopteran Ecology Package (BugsCEP). Umeå University, Umeå University.

Carlsen, P. 2017. Five-year numerical response of saproxylic beetles following a dead wood pulse left by moth outbreaks in northern Scandinavia. Master thesis. Norwegian University of Life Sciences, Aas, Norway.

Coombs, C., and G. Woodroffe. 1963. An experimental demonstration of ecological succession in an insect population breeding in stored wheat. Journal of Animal Ecology **32**:271-279.

Davis, B. N. K. 1989. The European distribution of insects on stinging nettles, Urtica dioica L.: A field survey. Bollettino di zoologia **56**:321-326.

Dedyukhin, S. V. 2016. Consortial associations of phytophagous beetles (Coleoptera: Chrysomeloidea, Curculionoidea) with plants in the east of the Russian Plain. Entomological Review **96**:679-700.

Echegaray, E. R., and R. A. Cloyd. 2013. Life History Characteristics of the Rove Beetle, <i>Dalotia coriaria</i> (Coleoptera: Staphylinidae) under Laboratory Conditions. **86**:145-154, 110.

ENHS. 1996. The Flora and Fauna of Exmoor National Park. Exmoor Natural History Society.

Fossest⊘L, K. O., and A. Sverdrup-Thygeson. 2009. Saproxylic beetles in high stumps and residual downed wood on clear-cuts and in forest edges. Scandinavian Journal of Forest Research **24**:403-416.

Frison, T. H. 1921. Antherophagus ochraceus Mels. In the Nests of Bumblebees. The American Naturalist **55**:188-192.

Gavrilović, B., and S. Ćurčić. 2013. The Diversity of the Family Chrysomelidae (Insecta: Coleoptera) of the Obedska Bara Special Nature Reserve (Vojvodina Province, Serbia), with Special Reference to the Host Plants. Acta Zoologica Bulgarica **65**:37-44.

Gossner, M. M., T. Lachat, J. Brunet, G. Isacsson, C. Bouget, H. Brustel, R. Brandl, W. W. Weisser, and J. Müller. 2013. Current Near-to-Nature Forest Management Effects on Functional Trait Composition of Saproxylic Beetles in Beech Forests. **27**:605-614.

Group, W. C. 2019. UK Beetle Recording.

Harde, K. W. 2000. A field guide in color to beetles. Silverdale Books, Prague, Czech Republic.

Heethoff, M., L. Koerner, R. A. Norton, and G. Raspotnig. 2011. Tasty but protected--first evidence of chemical defense in oribatid mites. J Chem Ecol **37**:1037-1043.

Hellqvist, M., and G. Lemdahl. 1996. Insect Assemblages and Local Environment in the Mediaeval Town of Uppsala, Sweden. Journal of Archaeological Science **23**:873-881.

Hirschberger, P. 1999. Larval population density affects female weight and fecundity in the dung beetle Aphodius ater. Ecological Entomology **24**:316-321.

Hjältén, J., R. Hägglund, T. Löfroth, J.-M. Roberge, M. Dynesius, J. J. B. Olsson, and Conservation. 2017. Forest restoration by burning and gap cutting of voluntary set-asides yield distinct immediate effects on saproxylic beetles. **26**:1623-1640.

Hjältén, J., F. Stenbacka, and J. Andersson. 2010. Saproxylic beetle assemblages on low stumps, high stumps and logs: Implications for environmental effects of stump harvesting. Forest Ecology and Management **260**:1149-1155.

Hofstertter, H., and F. Vega. 2015. Bark beetles: biology and ecology of native and invasive species. Elsevier Inc., London, UK.

Horák, J. 2011. Response of saproxylic beetles to tree species composition in a secondary urban forest area. Urban Forestry & Urban Greening **10**:213-222.

Horák, J. 2015. Suitability of biodiversity-area and biodiversity-perimeter relationships in ecology: a case study of urban ecosystems. Urban Ecosystems **19**:131-142.

Janssen, P., M. Fuhr, E. Cateau, B. Nusillard, and C. Bouget. 2017. Forest continuity acts congruently with stand maturity in structuring the functional composition of saproxylic beetles. Biological Conservation **205**:1-10.

Kalushkov, P., and O. Nedved. 2005. Genetically modified potatoes expressing Cry 3A protein do not affect aphidophagous coccinellids. Journal of Applied Entomology **129**:401-406.

Kočárek, P. 2003. Decomposition and Coleoptera succession on exposed carrion of small mammal in Opava, the Czech Republic. European Journal of Soil Biology **39**:31-45.

Koehler, F. 2000. Saproxylic beetles in nature forests of the northern Rhineland. Comparative studies on the saproxylic beetles of Germany and contributions to German nature forest research. Schrr. LÖBF/LAfAO NRW (Recklinghausen) **18**:1-351.

Kristofik, J., P. Masan, and Z. Sustek. 2001. Mites, beeltes and fleas in the enst of great reed walbler and reed walber. Biologia **56**:525-537.

Kvamme, T. 1982. New records of Noregain Coleoptera I. Species new to the fauna. Nowegain Journal of Entomology **29**:34-36.

Lapeva-Gjonova, A., and W. H. Rücker. 2011. Latridiidae and Endomychidae beetles (Coleoptera) from ant nests in Bulgaria. Mitteilungsblatt für Systematik und Taxonomie der Latridiidae **8**:5-8.

Lassauce, A., P. Anselle, F. Lieutier, and C. Bouget. 2012. Coppice-with-standards with an overmature coppice component enhance saproxylic beetle biodiversity: A case study in French deciduous forests. Forest Ecology and Management **266**:273-285.

Lott, D., G. Finch, and G. Price. 2011. A Provisional Atlas of the Carabidae of Leicestershire & Rutland The Leicestershire & Rutland Environmental Resources Centre, Leicestershire, UK.

Lupi, D., M. Colombo, and A. Zanetti. 2006. The rove beetles (Coleoptera Staphylinidae) of three horticultural farms in Lombardy (Northern Italy) Bollettino di Zoologia agraria e di Bachicoltura **38**:143-165.

Majzlan, O. 2015. Diversity of beetles (Coleoptera) in three types of orchards. Proceedings of the conference «Roubal’s Days I», Banská Bystrica **27**:15-25.

Memmott, J., N. Martinez, and J. Cohen. 2000. Predators, parasitoids and pathogens: species richness, trophic generality and body sizes in a natural food web. Journal of Animal Ecology **69**:1-15.

Montgomery, M., and S. Lyon. 1995. Natural enemies of Adelgids in North America: their prospect for biological control of Adelges tsugae.89-102.

Nagy, D. D., T. Magura, Z. Debnár, R. Horváth, and B. Tóthmérész. 2015. Shift of rove beetle assemblages in reforestations: Does nativity matter? Journal of Insect Conservation **19**:1075-1087.

Nikitsky, N., and D. Schigel. 2004. Beetles in polypores of the Moscow region: checklist and ecological notes. Entomologica Fennica **29**:6-22.

Nikitsky, N. B., and A. S. Ukrainsky. 2016. The ladybird beetles (Coleoptera, Coccinellidae) of Moscow Province. Entomological Review **96**:710-735.

Parmentier, T., S. Bouillon, W. Dekoninck, and T. Wenseleers. 2016. Trophic interactions in an ant nest microcosm: a combined experimental and stable isotope (δ13C/δ15N) approach. **125**:1182-1192.

Petrova, V., Z. Čudare, and R. Cibuļskis. 2006. Predators and herbivores beetles (coleoptera) naturally occurring on strawberry (Latvia). Acta Biol. Univ. Daugavp **6**:155-159.

Ponel, P., G. Coope, V. Andrieu-Ponel, and M. Reille. 1999. Coleopteran evidence for a mosaic of environments at high altitude in the eastern Pyre´ne´es, France, during the climatic transition between the Allerød and Younger Dryas. Journal of Quaternary Science **14**:169-174.

Priemé, A., T. B. Knudsen, M. Glasius, and S. Christensen. 2000. Herbivory by the weevil, Strophosoma melanogrammum, causes severalfold increase in emission of monoterpenes from young Norway spruce (Picea abies). Atmospheric Environment **34**:711-718.

Päivinen, J., P. Ahlroth, and V. Kaitala. 2002a. Ant-associated beetles of Fennoscandia and Denmark. Entomologica Fennica **13**:20-40.

Päivinen, J., P. Ahlroth, and V. Kaitala. 2002b. Ant-associated beetles of Fennoscandia and Denmark. Entomologica Fennica **13**.

Saska, P., and V. Jarosik. 2001. Laboratory study of larval food requirements in nine species of Amara (Celoptera: Carabidae). Plant Protection Science **37**:103-110.

Sawoniewicz, M. 2013. Beetles (Coleoptera) occurring in decaying birch (Betula spp.) wood in the Kampinos National Park. Forest Research Papers **74**.

Schigel, D. S., T. Niemelä, M. Similä, J. Kinnunen, and O. Manninen. 2004. Polypores and associated beetles of the North Karelian Biosphere Reserve, eastern Finland. Karstenia **44**:35-56.

Seibold, S., R. Brandl, J. Buse, T. Hothorn, J. Schmidl, S. Thorn, and J. Muller. 2015. Association of extinction risk of saproxylic beetles with ecological degradation of forests in Europe. Conserv Biol **29**:382-390.

Sloggett, J., and M. Majerus. 2000. Aphid-mediated coexistence of ladybirds (Coleoptera: Coccinellidae) and the wood ant Formica rufa: seasonal effects, interspecific variability and the evolution of a coccinellid myrmecophile. Oikos **89**:345-359.

Sobek, S., I. Steffan-Dewenter, C. Scherber, and T. Tscharntke. 2009. Spatiotemporal changes of beetle communities across a tree diversity gradient. Diversity and Distributions **15**:660-670.

Stan, M. 2003. Tachyporus dispar (Paykull, 1789) and Haploglossa nidicola (Fairmaire, 1852) (Coleoptera: Staphylindae) Two newmentions in the Staphylinid fauna of Romania. Muzeul National de Istorie Natural:187-192.

Stokland, J. N., and E. Meyke. 2008. The saproxylic database: an emerging overview of the biological diversity in dead wood. Revue d'écologie **63**:37-48.

Sunderland, K. D., and S. L. Sutton. 1980. A Serological Study of Arthropod Predation on Woodlice in a Dune Grassland Ecosystem. Journal of Animal Ecology **49**:987-1004.

Sutherland, K., and P. Vickerman. 1980. Aphid feeding by some polyphagous predators in relation to aphid density in cereal fields. Journal of Applied Ecology **17**:389-396.

Sweeney, J., R. S. Anderson, R. P. Webster, and R. Neville. 2012. First Records of Orchestes Fagi (L.) (Coleoptera: Curculionidae: Curculioninae) in North America, with a Checklist of the North American Rhamphini. The Coleopterists Bulletin **66**:297-304.

Turis, P., and Ľ. Vidlička. 2013. Relationship of animals to the cyclamen Cyclamen fatrense Halda et Soják: pollinators, consumers and occasional visitors. Biologia **68**.

Veith, M., N. J. Oldham, K. Dettner, J. M. Pasteels, and W. Boland. 1997. Biosynthesis of defensive allomones in leaf beetle larvae: stereochemistry of Salicylalcohol oxidation in Phratora vitellinae and comparison of enzyme substrate and stereospecificity with alcohol oxidases from several iridoid producing leaf beetles. Journal of Chemical Ecology **23**:429-443.

Vickerman, G. P., and K. D. Sunderland. 1977. Some Effects of Dimethoate on Arthropods in Winter Wheat. Journal of Applied Ecology **14**:767-777.

Vindstad, O. P. L., S. Schultze, J. U. Jepsen, M. Biuw, L. Kapari, A. Sverdrup-Thygeson, and R. A. Ims. 2014. Numerical Responses of Saproxylic Beetles to Rapid Increases in Dead Wood Availability following Geometrid Moth Outbreaks in Sub-Arctic Mountain Birch Forest. PLoS One **9**:e99624.

Wallace, H. R. 1953. The Ecology of the Insect Fauna of Pine Stumps. The Journal of Animal Ecology **22**:154.

Yeo, P. F. 1972. Miscellaneous notes on pollination and pollinators. Journal of Natural History **6**:667-686.
